# Stage-associated immunoproteomic profiling of serum autoantibody-captured retinal antigens in age-related macular degeneration

**DOI:** 10.3389/fimmu.2026.1824524

**Published:** 2026-07-08

**Authors:** Hao Lin, Peter Wolfrum, Lia F. Kempf, Dominik Wolters, Natarajan Perumal, Christina A. Korb, Franz H. Grus

**Affiliations:** Experimental and Translational Ophthalmology, Department of Ophthalmology, University Medical Center of the Johannes Gutenberg-University Mainz, Mainz, Germany

**Keywords:** age-related macular degeneration (AMD), antigen microarray, anti-retinal antibodies, MS, retinal antigen targets, serum, tears

## Abstract

**Introduction:**

Increasing evidence suggests that autoimmune responses are involved in the pathogenesis of age-related macular degeneration (AMD). This study aimed to characterize changes in serum autoantibody-captured retinal antigens in patients with different AMD subtypes, in order to gain further insight into the immunological mechanisms associated with AMD progression.

**Methods:**

Serum samples were collected from healthy controls (CTRL, n = 15) and patients with early AMD (n = 15), intermediate AMD (Int. AMD, n = 15), late AMD (n = 15), neovascular AMD with type 1 macular neovascularization (nAMD type 1, n = 10), and neovascular AMD with type 2 macular neovascularization (nAMD type 2, n = 10). Serum antibodies were isolated using Protein G magnetic beads and incubated with porcine retinal lysates to capture antigen–antibody complexes. After elution, the captured retinal antigens were subjected to proteomic analysis.

**Results:**

Mass spectrometry identified 91 differentially captured retinal antigen targets across AMD subtypes. Compared with controls, 11, 9, 11, 18, and 22 retinal antigen targets were significantly increased in serum samples from patients with early AMD, intermediate AMD, late AMD, nAMD type 1, and nAMD type 2, respectively. Conversely, 14, 11, 9, 10, and 20 retinal antigen targets were decreased in the corresponding AMD subtypes, respectively (FDR < 0.05; p < 0.05). Selected mass spectrometryderived retinal antigen targets were further evaluated in serum and tear samples using antigen microarray analysis. Consistent with the proteomics results, anti-ATP5A1, anti-HIST2H2AA3, and anti-PFKM autoantibody levels were significantly increased in tears from patients with nAMD type 2 (p = 0.03, p = 0.01, and p = 0.04, respectively). In addition, anti-FTH1 autoantibody levels were decreased in serum from patients with nAMD type 1.

**Discussion:**

Comprehensive screening of serum autoantibody-captured retinal antigens across AMD stages, together with pathway enrichment and STRING network analyses, revealed subtype-specific immune alterations. These findings provide new insights into the immunopathological features of AMD.

## 1 Introduction

Age-related macular degeneration (AMD) is a progressive retinal disorder and one of the leading causes of vision loss in individuals over the age of 55 (1). Although early and intermediate stages are often asymptomatic, late-stage AMD can lead to severe and irreversible visual impairment (2).

The eye is an immune-privileged organ, a specialized state that protects ocular tissues from excessive inflammation and immune-mediated damage (3). However, this unique immune privilege can also render the eye vulnerable to autoimmune attack when immunological tolerance is disrupted (4). Recent studies have increasingly reported an involvement of autoimmunity in the progression of AMD (5–7). In our previous work, we demonstrated distinct IgG autoantibody patterns against retinal antigens in patients with neovascular AMD compared with healthy individuals (8). AMD serum samples showed both increased and decreased reactivity in retinal antigens across different molecular weight ranges, indicating alterations in autoimmune responses (9). In addition, elevated autoantibody levels against specific retinal antigens, such as glial fibrillary acidic protein, have been observed in AMD patients relative to controls (8). Despite these findings, the relevance and functional role of anti-retinal autoantibodies in AMD remain unclear. It is still uncertain whether these antibodies actively target specific retinal components, such as the retinal pigment epithelium (RPE), thereby contributing to AMD pathogenesis, or whether they emerge as secondary by-products of disease progression.

Dry and neovascular AMD represent biologically distinct entities with different underlying pathogenic mechanisms (2). At present, it remains unclear how autoantibody reactivity against retinal antigens differs across the various stages and subtypes of AMD. A systematic characterization of anti-retinal autoantibody responses across disease stages may help identify previously unrecognized immunological features of AMD and provide new insights into disease-associated immune alterations during AMD progression. Such findings may also improve our understanding of dynamic changes in serum and tear autoantibody profiles in relation to AMD stage and neovascular subtype.

In this study, we aimed to comprehensively screen serum autoantibody-captured retinal antigen targets in patients with different AMD subtypes and to characterize stage-associated patterns of anti-retinal autoantibody reactivity. A selected subset of candidate targets was further assessed in serum and tear samples using a targeted antigen microarray platform. By integrating immunoproteomic screening with targeted exploratory evaluation, this study may provide insight into stage-associated immune alterations in AMD and identify candidate retinal antigen targets for future validation.

## 2 Materials and methods

### 2.1 Participants selection and AMD grading

Ethical approval for the study protocol and design was obtained from the local Ethics Committee of the State Medical Association of Rhineland-Palatinate, Germany (Landesärztekammer Rheinland-Pfalz; approval number: 2020-15188). The study was conducted in accordance with the Declaration of Helsinki. All participants were fully informed about the study’s aims, procedures, potential risks, and data protection policies, and written informed consent was obtained from each subject prior to enrollment. Subjects were recruited from the Department of Ophthalmology at the University Medical Center of the Johannes Gutenberg University Mainz.

**Figure 1:**
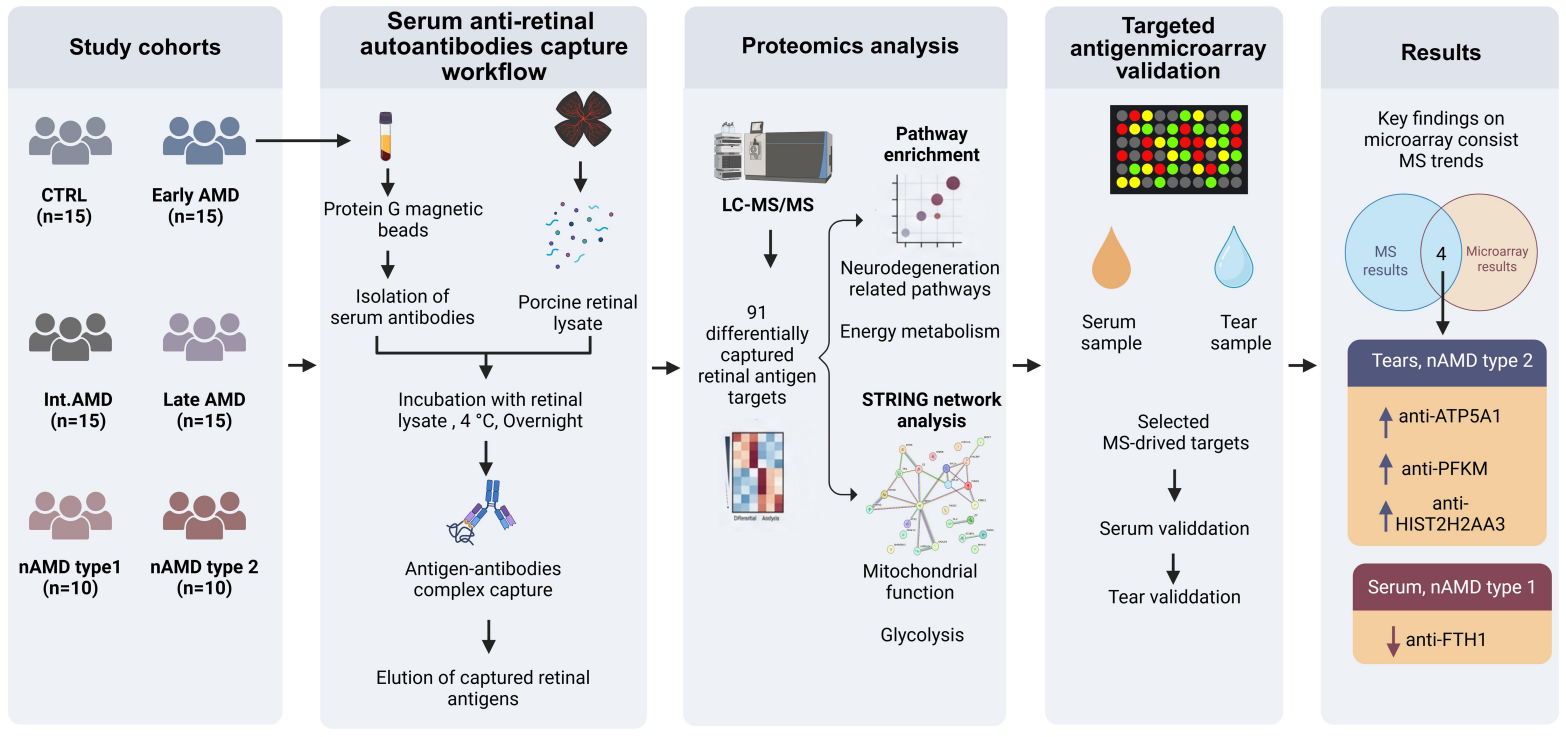
Overview of the experimental workflow for analyzing retinal antigen profiles captured by serum autoantibodies from patients with different stages of AMD. Antibodies isolated from AMD patient serum were captured on Protein G magnetic beads and incubated overnight with porcine retinal lysates. Unbound retinal antigens were washed away, and antibody-bound antigens were subsequently subjected to MS analysis. Selected differentially expressed retinal antigen targets identified by MS were further evaluated and validated in serum and tear samples from AMD patients using antigen microarray technology. The image was created using BioRender.com (https://biorender.com/, accessed on 14. June 2026).

A total of 80 subjects were recruited between November 2020 and March 2021. The cohort included 15 subjects with early AMD, 15 subjects with intermediate AMD, 15 subjects with late AMD, and 20 treatment-naïve neovascular AMD patients, consisting of 10 patients with type I neovascularization and 10 patients with type II neovascularization. Additionally, 15 healthy individuals without clinical signs of retinal disease served as controls (**Figure 1**).

AMD grading was performed according to a previously published consensus classification system (10). Detailed descriptions of the grading criteria, diagnostic procedures, and participant eligibility criteria are provided in the **Supplementary Methods**. Participant demographics and clinical characteristics are summarized in **Table 1**.

**Table 1:** Descriptive study cohort characteristics.

Group	N	Mean age	SD	Interquartile range	Male	Female
CTRL	15	66.0	10.0	58.0 – 73.8	9	6
Early AMD	15	67.6	11.5	61.0 – 75.8	7	8
Int. AMD	15	77.2	7.7	72.5 – 82.4	9	6
Late AMD	15	75.4	7.0	71.5 – 80.2	5	10
nAMD type 1	10	78.9	7.0	76.6 – 82.7	4	6
nAMD type 2	10	70.4	9.2	66.7 – 77.3	6	4
Total	80	72.3	10.0	65.5 – 79.6	40	40

### 2.2 Serum and tear sample collection

Blood samples were collected before any medical treatment. Participants were instructed to fast for approximately 8 h before venipuncture. Blood samples were collected into serum separator tubes containing clot activator and separation gel. After clotting, the samples were centrifuged at 1, 000 × g for 10 min. The serum supernatant was then aliquoted into sterile cryovials and immediately stored at −80 °C until further processing.

As described in our previous study (11), tear samples were collected from one eye of each participant using Schirmer strips. In patients with bilateral AMD, the eye corresponding to the assigned AMD stage was selected. If both eyes met the same staging criteria, the right eye was selected; if the right eye was not eligible, the left eye was used. No topical anesthetic eye drops were applied during tear collection, as local anesthetics may suppress tear secretion. The collected Schirmer strips (Schirmer Tear Test; Optitech Eyecare, Uttar Pradesh, India) were placed in safety-lock microcentrifuge tubes and immediately stored at −80 °C until use.

For tear protein extraction, 300 µL of phosphate-buffered saline (PBS) was added to each Schirmer strip, followed by incubation with agitation using an IntelliMixer for 3 h at 4 °C.

### 2.3 Porcine retinal tissue extraction and homogenization

Retinal extraction and homogenization were performed following previously established protocols (12, 13). The use of domestic pig by-products for scientific research was approved by the Mainz-Bingen district administration (code: DE 07 315 0006 21). Fresh porcine eye bulbs (Sus scrofa domestica, n = 20) were obtained from a local slaughterhouse (Landmetzgerei Harth, Stadecken-Elsheim, Germany). Retinas were isolated and transferred into 2-ml screw-cap tubes containing ceramic beads and 1 ml of T-PER extraction buffer (Thermo Fisher Scientific, Waltham, MA, USA). Tissue homogenization was carried out using a Bullet Blender^®^ Storm 24 (Next Advance, Troy, NY, USA) as described previously (14, 15). Homogenates were centrifuged at 14, 000 g for 15 min at 4 °C, and the resulting protein-containing supernatant was buffer-exchanged into 100 μl PBS using a 3-kDa Amicon^®^ filtration unit (Millipore, Burlington, MA, USA). The samples were subsequently used for further processing.

### 2.4 Identification of autoantigens

The aim of this study was to identify novel autoantibody targets using MS-AMIDA (mass spectrometry–based identification of antigens by autoantibodies) (16). For the discovery phase, serum samples from each clinical group were analyzed as three independent pooled replicates. Each pooled replicate was prepared by combining equal amounts of serum from multiple subjects within the same group. Antibodies were first isolated from pooled serum samples of each group using protein A/G affinity columns. Purified IgG (100 µg) was immobilized on protein G magnetic beads and incubated overnight at 4 °C with 5 mg of porcine retinal lysate to capture the corresponding anti-retinal autoantibodies. After extensive washing, bound antigens were eluted, digested with trypsin, purified using SOLAμ™ HRP SPE plates (Thermo Fisher Scientific, Waltham, MA, USA), and analyzed on a hybrid Orbitrap linear ion trap mass spectrometer (LTQ Orbitrap XL, Thermo Fisher Scientific, USA).

### 2.5 Mass spectrometry-based proteomics analysis

The reverse phase before peptide analysis was performed using an EASY-nLC 1200 system (Thermo Fisher Scientific, Rockford, USA). The peptides were separated into an analytical column (75 μm × 50 cm, nanoViper, C18, 2 μm, 100 Å) (Thermo Fisher Scientific, Rockford, USA). Solvent A was 0.1% formic acid in water and solvent B was 0.1% formic acid in 80% acetonitrile. The gradient elution of the peptides took place within 120 min as follows: 5% - 30% B (0–90 min), 30-100% B (90–100 min) and 100% B (100–120 min). After separation, the peptides were ionized with a nanoelectrospray ionization source and transferred to the mass spectrometer. The LTQ-Orbitrap was used in positive ionization and data-dependent acquisition mode to automatically switch between Orbitrap-MS acquisition and LTQ-MS/MS acquisition.

The full-scan MS spectra (from m/z 300 to 2000) were recorded in the Orbitrap with a resolution of 30000 to 400 m/z and a target gain control setting of 1 × 10^6^ ions. For the mass lock option, polydimethylcyclosiloxane ions m/z 445.120025 were used for internal calibration. The dynamic exclusion mode was defined as follows: number of repetitions = 2, repetition time = 30 s, exclusion list size = 100, exclusion time = 90 s, and exclusion mass width = ± 20 ppm. The five most intense precursor ions were selected for further fragmentation in the collision-induced decay fragmentation (CID) ion trap using the normalized collision energy defined to be 35%.

### 2.6 MS-based protein identification, LFQ quantification, and data preprocessing

MS data were analyzed using MaxQuant software version 1.6.1.0 (Max Planck Institute for Biochemistry, Martinsried, Germany). Tandem MS spectra were searched against the UniProt Human and Sus scrofa databases using standard search parameters, including a peptide mass tolerance of ±30 ppm, a fragment ion tolerance of 0.5 Da, tryptic digestion with a maximum of two missed cleavages, carbamidomethylation as a fixed modification, and N-terminal acetylation and methionine oxidation as variable modifications.

For the MS-based discovery phase, MaxQuant output files were further processed using Perseus software version 1.6.13.0 (Max Planck Institute for Biochemistry, Martinsried, Germany). LFQ intensity values were log_2_-transformed before downstream analysis. Protein groups annotated as contaminants or reverse hits were removed. Proteins were retained only if they were quantified in at least 70% of samples within at least one clinical group. Missing LFQ intensity values were imputed from a normal distribution using a width of 0.3 and a downshift of 1.8.

### 2.7 Antigen microarrays

Antigen microarrays were used to analyze and compare autoantibody profiles in serum and tear fluid from patients with different AMD stages and controls. Autoantigen candidates identified from *de novo* MS screening were further validated using this approach. Raw fluorescence data were normalized (e.g., Z-score algorithm) to enable comparison of spot intensities and to identify antigens that discriminate AMD patients from controls.

Commercially obtained antigens (**Supplementary Table 1**) were spotted in triplicates onto nitrocellulose-coated slides (Oncyte, Grace Bio Labs, USA) using a non-contact microarray spotter (sciFLEXARRAYER S3, Scienion, Germany). Spot quality and volume were monitored by integrated camera software. After air-drying (~12 h), slides were assembled in FAST frame hybridization chambers and blocked for 1 h (Super G Blocking Reagent). Slides were washed three times in PBS-T (PBS + 0.5% Tween-20, 10 min each) before incubation with serum (1:150 dilution) or tear samples (70 μL + 30 μL PBS) for 16 h at 4 °C with gentle agitation.

Following three additional PBS-T washes, slides were incubated with fluorescently labeled anti-human IgG (H+L, 1:500, Jackson ImmunoResearch) for 1 h in the dark, washed again, rinsed in ultrapure water, and dried under vacuum. Fluorescence signals were acquired using a confocal microarray scanner (SensoSpot, Sensovation, Germany) and exported as 16-bit TIFF files. PBS incubations served as negative controls.

Raw signal extraction was performed with Imagene software (BioDiscovery), excluding technically flawed spots. Background-corrected mean intensities were normalized using a constant scaling factor per subarray, and technical replicates were averaged.

### 2.8 Statistical analysis

Detailed procedures for data preprocessing, filtering, normalization, statistical testing, multiple-testing correction, and bioinformatics analyses are provided in the **Supplementary Methods**; briefly, MS-derived LFQ data were analyzed in Perseus using log_2_ transformation, valid-value filtering, imputation, and pairwise AMD subgroup-versus-CTRL comparisons with Benjamini–Hochberg FDR correction, whereas targeted antigen microarray data were analyzed using Kruskal-Wallis ANOVA followed by Mann-Whitney U tests, and GO/pathway enrichment and protein–protein interaction analyses were performed using Bioinformatics.com.cn and STRING, respectively.

## 3 Results

### 3.1 Changes in serum autoantibody-captured retinal antigens across different AMD subtypes compared with controls

**Figure 2:**
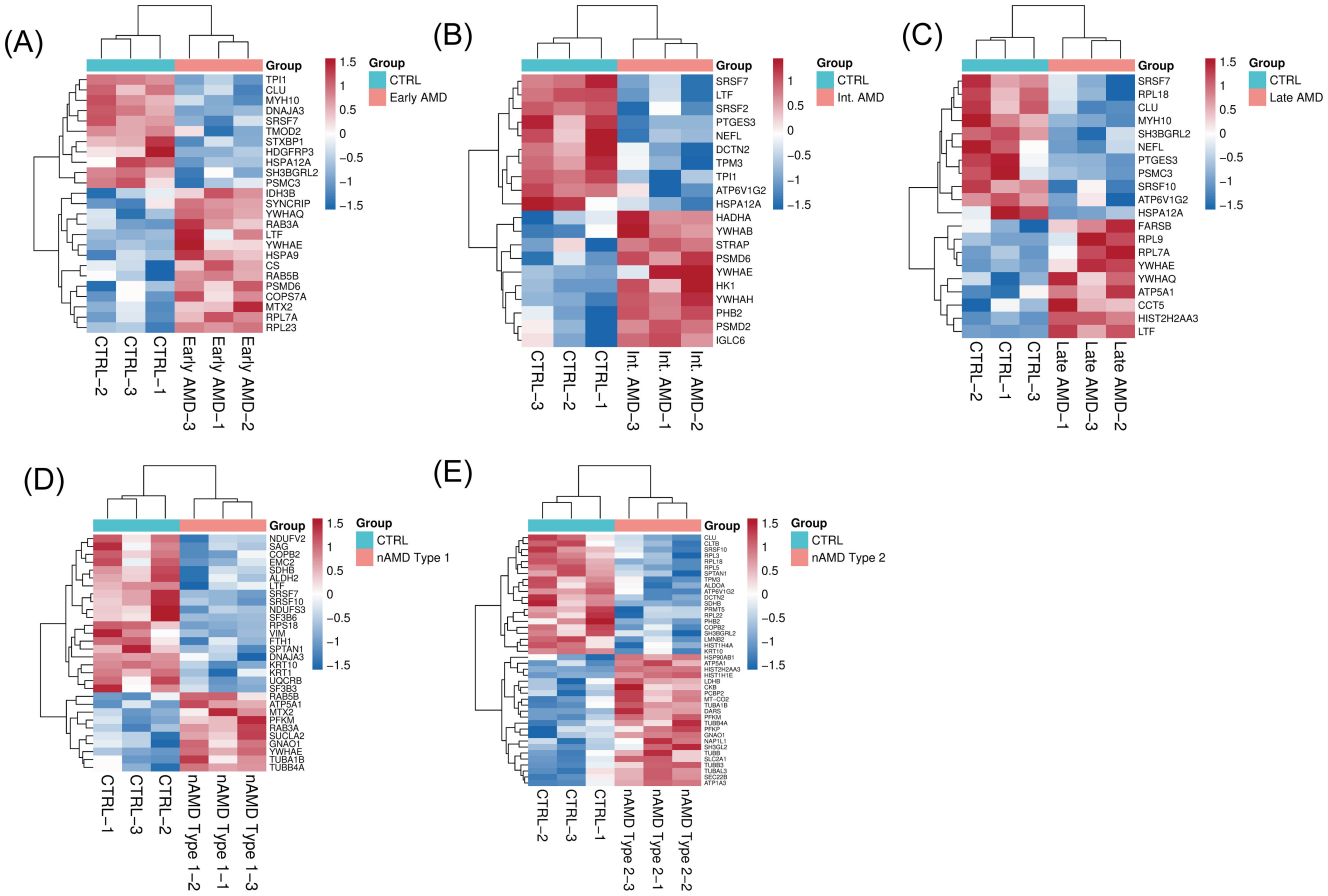
Heatmaps illustrate differential retinal antigen profiles captured by serum autoantibodies from patients at various AMD stages compared with CTRL. Up-regulated proteins are shown in red and down-regulated proteins in blue, with color intensity reflecting the magnitude of the log_2_-fold change. For each group, columns 1–3 represent three independent pooled serum replicates. **(A)** Early AMD vs. CTRL; **(B)** Int. AMD vs. CTRL; **(C)** Late AMD vs. CTRL; **(D)** nAMD Type 1 vs. CTRL; **(E)** nAMD Type 2 vs. CTRL. Differentially enriched retinal antigens were selected based on statistical significance and fold-change criteria, including p < 0.05 and FDR < 0.05.

Mass spectrometry analysis of serum autoantibody-captured retinal antigens identified 91 retinal antigen targets. Differentially captured retinal antigens were defined using both a nominal statistical threshold of p < 0.05 from two-tailed Student’s t-tests and a Benjamini–Hochberg false discovery rate threshold of FDR < 0.05 for multiple-testing correction in group-wise comparisons. Compared with the CTRL group, increased abundance of captured retinal antigen targets was observed for 11 proteins in early AMD, 9 in intermediate AMD, 11 in late AMD, 18 in nAMD type 1, and 22 in nAMD type 2. In contrast, 14, 11, 9, 10, and 20 captured retinal antigen targets, respectively, showed decreased abundance relative to CTRL. **Supplementary Data Sheet 1** provides the log_2_-transformed normalized intensities, log_2_ fold changes, nominal p values, and adjusted p values for the significantly altered retinal antigen targets shown in the heatmaps. Heatmaps in **Figures 2A–E** display the row-wise Z-score-normalized abundance patterns of these differentially captured retinal antigens.

### 3.2 Pathway enrichment analysis of differential retinal autoantigens identified in serum from AMD subtypes compared with controls

**Figure 3:**
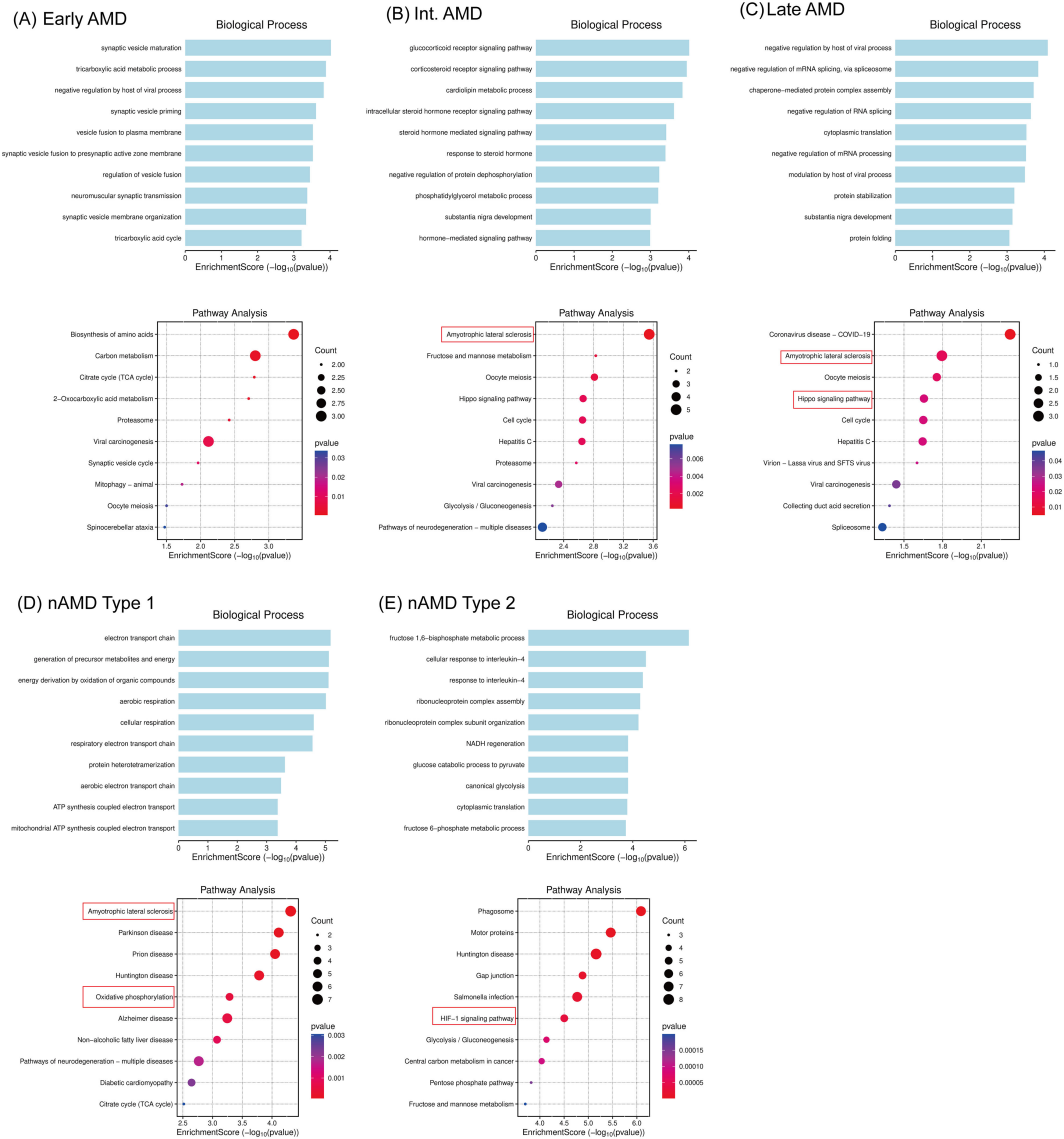
Functional enrichment analysis of differentially enriched retinal antigens captured by serum autoantibodies from patients with different stages of AMD. The upper panels show enriched Gene Ontology biological process terms, and the lower panels show enriched pathways. The x-axis represents the enrichment score, calculated as −log_10_(p value). Bubble size indicates the number of proteins involved, while color represents the corresponding p value. **(A)** Early AMD vs. CTRL; **(B)** Int. AMD vs. CTRL; **(C)** Late AMD vs. CTRL; **(D)** nAMD Type 1 vs. CTRL; **(E)** nAMD Type 2 vs. CTRL.

Functional enrichment analysis demonstrated subtype-specific biological patterns among the differentially captured retinal antigen targets (**Figure 3**). Early and intermediate AMD were mainly associated with synaptic, metabolic, proteasomal, and neurodegeneration-related pathways (**Figures 3A, B**). whereas late non-neovascular AMD showed enrichment in RNA processing, translation, protein folding, and spliceosome-related processes (**Figure 3C**). In nAMD type 1, the enrichment profile was dominated by mitochondrial respiration, oxidative phosphorylation, ATP synthesis, and neurodegeneration-associated pathways (**Figure 3D**). By contrast, nAMD type 2 was characterized by glycolytic and carbon metabolism, inflammatory signaling, ribonucleoprotein complex assembly, and cytoskeletal or transport-related pathways (**Figure 3E**). Overall, these results indicate distinct functional signatures of serum autoantibody-captured retinal antigen targets across AMD subtypes.

### 3.3 Protein–protein interaction network analysis of differentially captured retinal antigen targets

**Figure 4:**
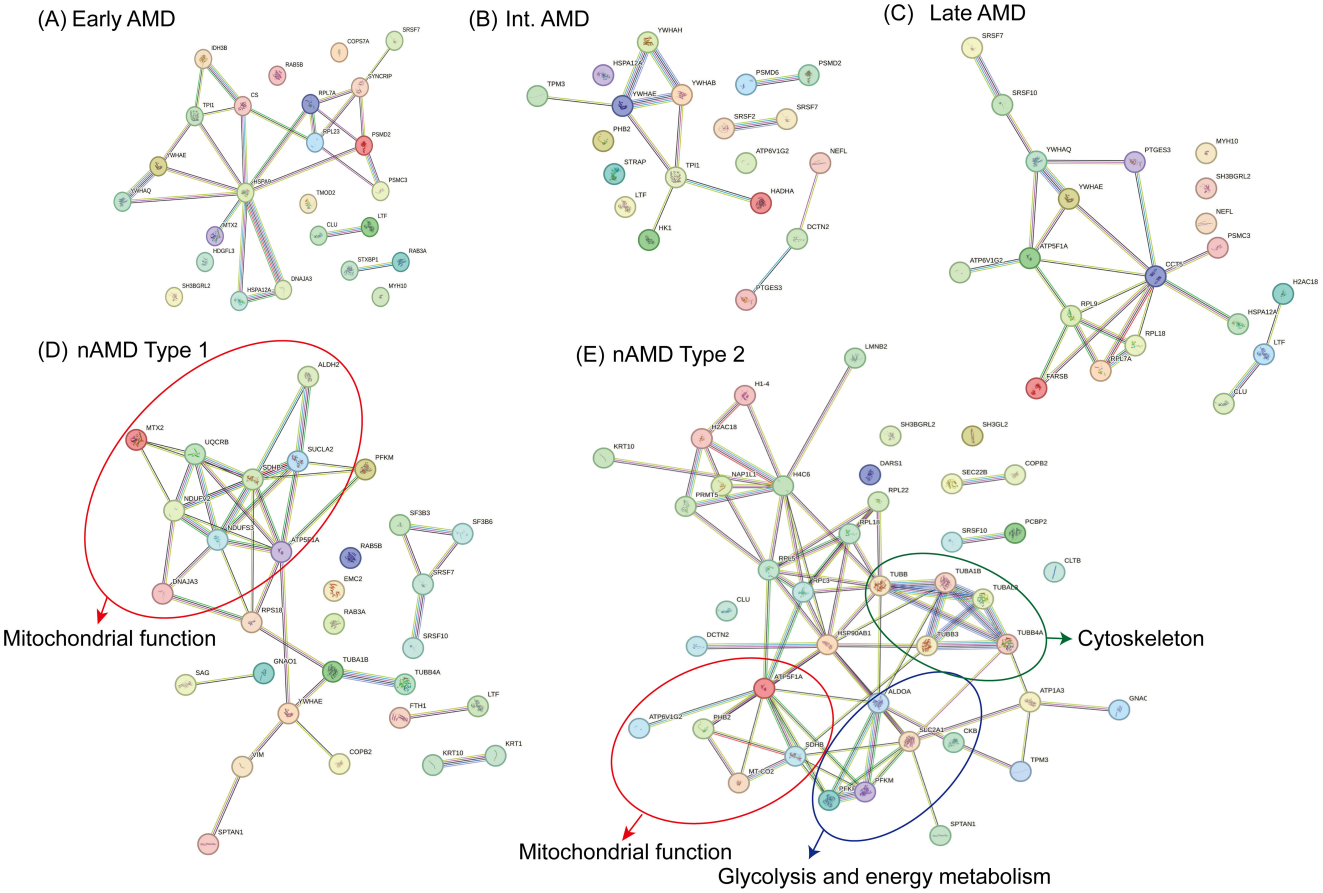
STRING-based protein–protein interaction network analysis of differentially enriched retinal antigens captured by serum autoantibodies from patients with different stages of AMD. Networks were generated for the differentially enriched retinal antigens identified in each AMD group compared with CTRL. Nodes represent proteins, and edges indicate known or predicted functional interactions. **(A)** Early AMD; **(B)** Int. AMD; **(C)** Late AMD; **(D)** nAMD Type 1; **(E)** nAMD Type 2. Representative functional clusters, including mitochondrial function, glycolysis and energy metabolism, and cytoskeleton-related proteins, are highlighted.

To further assess functional relationships among the differentially captured retinal antigen targets, protein–protein interaction networks were generated using STRING. Early and intermediate AMD displayed relatively limited network connectivity (**Figures 4A, B**). In late AMD, the connected nodes were mainly associated with RNA processing, translation, and protein homeostasis (**Figure 4C**), in line with the pathway enrichment results.

A more prominent interaction module was observed in nAMD type 1 (**Figure 4D**), where multiple captured retinal antigen targets clustered around mitochondrial function and energy metabolism. In nAMD type 2 (**Figure 4E**), the STRING network revealed several functional modules, including mitochondrial function, glycolysis and energy metabolism, and cytoskeletal or transport-related proteins. Overall, the networks showed AMD subtype-specific interaction patterns.

### 3.4 Dynamic changes in MS-derived retinal antigen targets across AMD progression and targeted microarray validation

**Figure 5:**
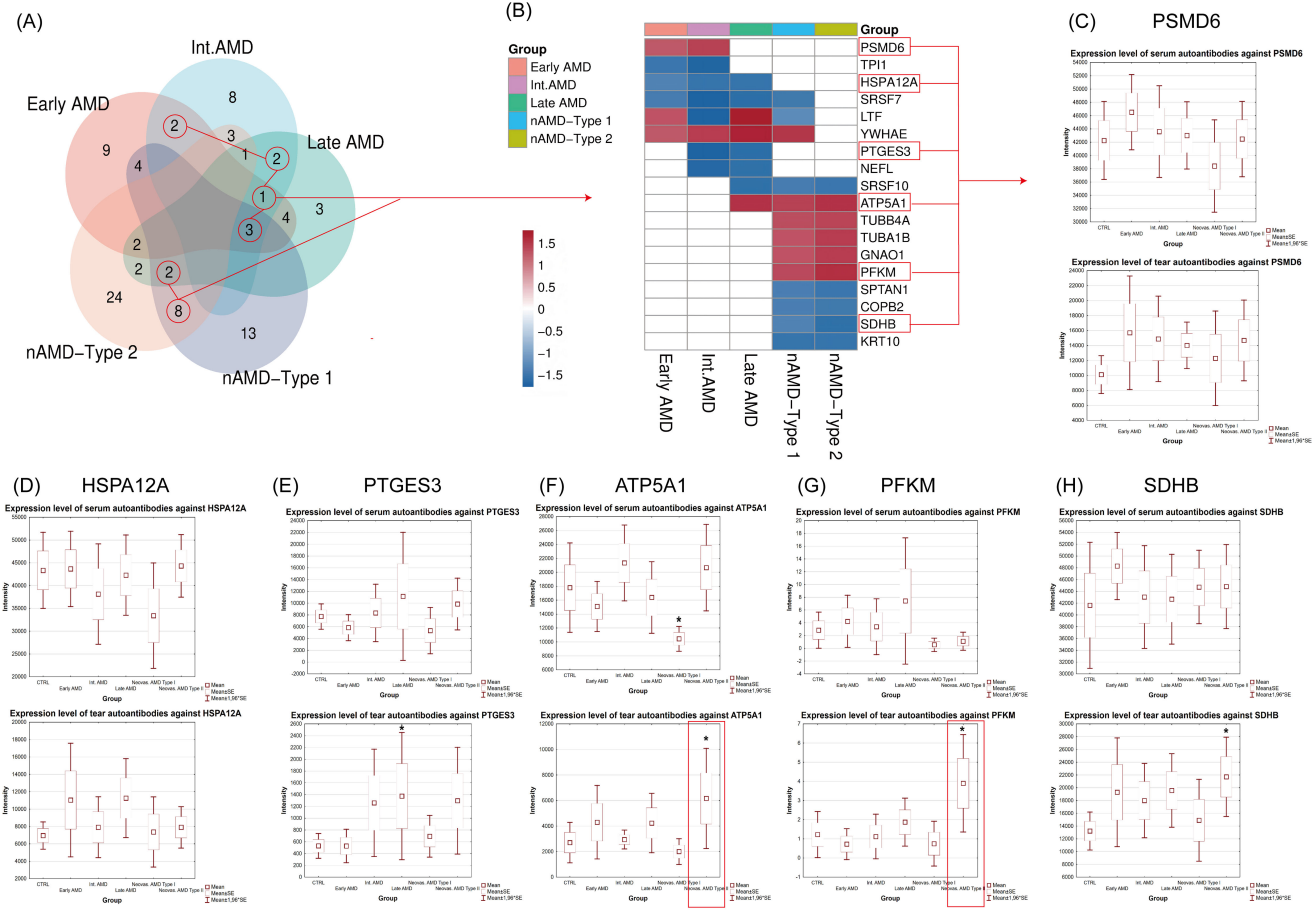
Selection and validation of candidate retinal antigens recognized by serum and tear autoantibodies in patients with different stages of AMD. **(A)** Venn diagram showing shared and stage-specific differentially enriched retinal antigens captured by serum autoantibodies across AMD subgroups. **(B)** Heatmap showing the relative abundance of selected candidate retinal antigens across AMD stages. Red indicates increased abundance and blue indicates decreased abundance. Candidate antigens selected for further validation are highlighted with red boxes. **(C–H)** Antigen microarray validation of serum and tear autoantibody reactivity against selected retinal antigens, including PSMD6, HSPA12A, PTGES3, ATP5A1, PFKM, and SDHB, in CTRL and AMD subgroups. Data are presented as mean ± SE. *p < 0.05.

Based on the candidate retinal antigen targets identified by MS, Venn diagram analysis was performed to explore the dynamic changes in serum autoantibody-captured retinal antigens across AMD progression (**Figures 5A, B**), from early AMD to nAMD type 2. PSMD6 and TPI1 were significantly altered in both early and intermediate AMD. HSPA12A showed changes from early to late non-neovascular AMD, whereas SRSF7, LTF, and YWHAE were altered across a broader disease spectrum, extending from early AMD to nAMD type 1. PTGES3 and NEFL showed changes from intermediate AMD to late non-neovascular AMD. SRSF10 and ATP5A1 were altered from late non-neovascular AMD to nAMD type 2. In contrast, eight retinal antigen targets, including PFKM and SDHB, were altered only in neovascular AMD. These findings suggest that serum autoantibody-captured retinal antigen targets display distinct stage-associated patterns during AMD progression.

To further evaluate selected dynamically altered retinal antigen targets, targeted microarray analysis was performed to quantitatively assess selected corresponding autoantibody responses in tear and serum samples (**Figures 5C–H**). Among the MS-derived candidates, anti-PFKM and anti-ATP5A1 autoantibody levels showed consistent changes with the MS-based screening results, with significantly increased levels detected in tears from patients with nAMD type 2 (p = 0.04 and p = 0.03, respectively; **Figures 5F, G**). Moreover, anti-SDHB autoantibody levels were also significantly increased in tears from patients with nAMD type 2 (p = 0.02; **Figure 5H**). Anti-PTGES3 autoantibody levels were significantly increased in tears from patients with intermediate AMD (p = 0.03; **Figure 5E**), representing a microarray-detected tear autoantibody change that was not concordant with the serum MS-based screening pattern. These findings suggest partial concordance between MS-based screening and targeted microarray analysis, while also highlighting that some tear autoantibody changes may differ from serum MS-derived patterns.

### 3.5 Targeted microarray validation of neovascular AMD-associated autoantibody responses

**Figure 6:**
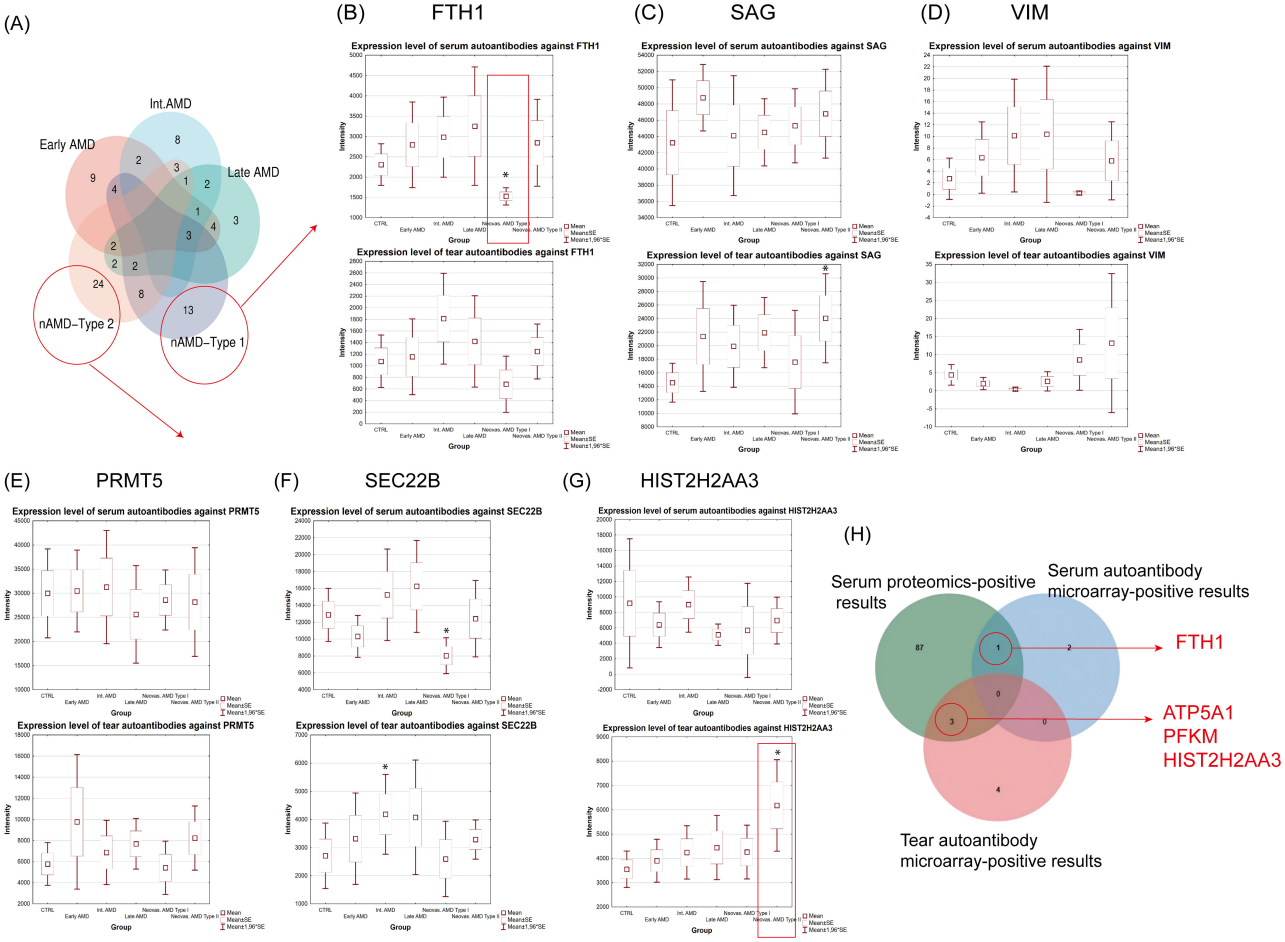
Validation and overlap analysis of selected candidate retinal antigens recognized by serum and tear autoantibodies in patients with different stages of AMD. **(A)** Venn diagram showing shared and stage-specific differentially enriched retinal antigens captured by serum autoantibodies across AMD subgroups. **(B–G)** A subset of candidate retinal antigen targets identified by serum autoantibody-based proteomic profiling, including FTH1, SAG, VIM, PRMT5, SEC22B, and HIST2H2AA3, was selected for further validation by antigen microarray analysis in serum and tear samples from CTRL and AMD subgroups. Data are presented as mean ± SE, with whiskers indicating 1.96 × SE.*p < 0.05. **(H)** Venn diagram showing the overlap among serum proteomics-positive results, serum autoantibody microarray-positive results, and tear autoantibody microarray-positive results. The antigen microarray analysis was performed as a targeted validation of selected MS-derived retinal antigen targets. Candidate antigens supported by multiple datasets are highlighted in red.

In the targeted microarray analysis, changes in anti-retinal autoantibody levels were more frequently observed in neovascular AMD. Based on the MS-derived retinal antigen targets and previous reports, selected retinal antigens associated with neovascular AMD were further evaluated by microarray analysis to quantify the corresponding autoantibody responses in tear and serum samples (**Figure 6A**).

Consistent with the MS-based screening results (**Figures 6B–G**), anti-FTH1 autoantibody levels were significantly decreased in the serum of patients with nAMD type 1 (p = 0.04; **Figure 6B**), whereas anti-HIST2H2AA3 autoantibody levels were significantly increased in tears from patients with nAMD type 2 (p = 0.01; **Figure 6G**). In addition, anti-SAG autoantibody levels were significantly increased in tears from patients with nAMD type 2 (p = 0.008; **Figure 6C**), and anti-SEC22B autoantibody levels were significantly increased in tears from patients with intermediate AMD (p = 0.03; **Figure 6F**).

Overall, targeted microarray analysis identified seven significantly altered anti-retinal autoantibody responses in tear samples and three in serum samples. Among these, four targets showed changes concordant with the MS-based screening results: anti-FTH1 autoantibodies in nAMD type 1 serum, and anti-ATP5A1, anti-PFKM, and anti-HIST2H2AA3 autoantibodies in nAMD type 2. These findings provide partial validation of selected MS-derived retinal antigen targets (**Figure 6H**).

### 3.6 Correlation of selected anti-retinal autoantibody responses between serum and tears

**Figure 7:**
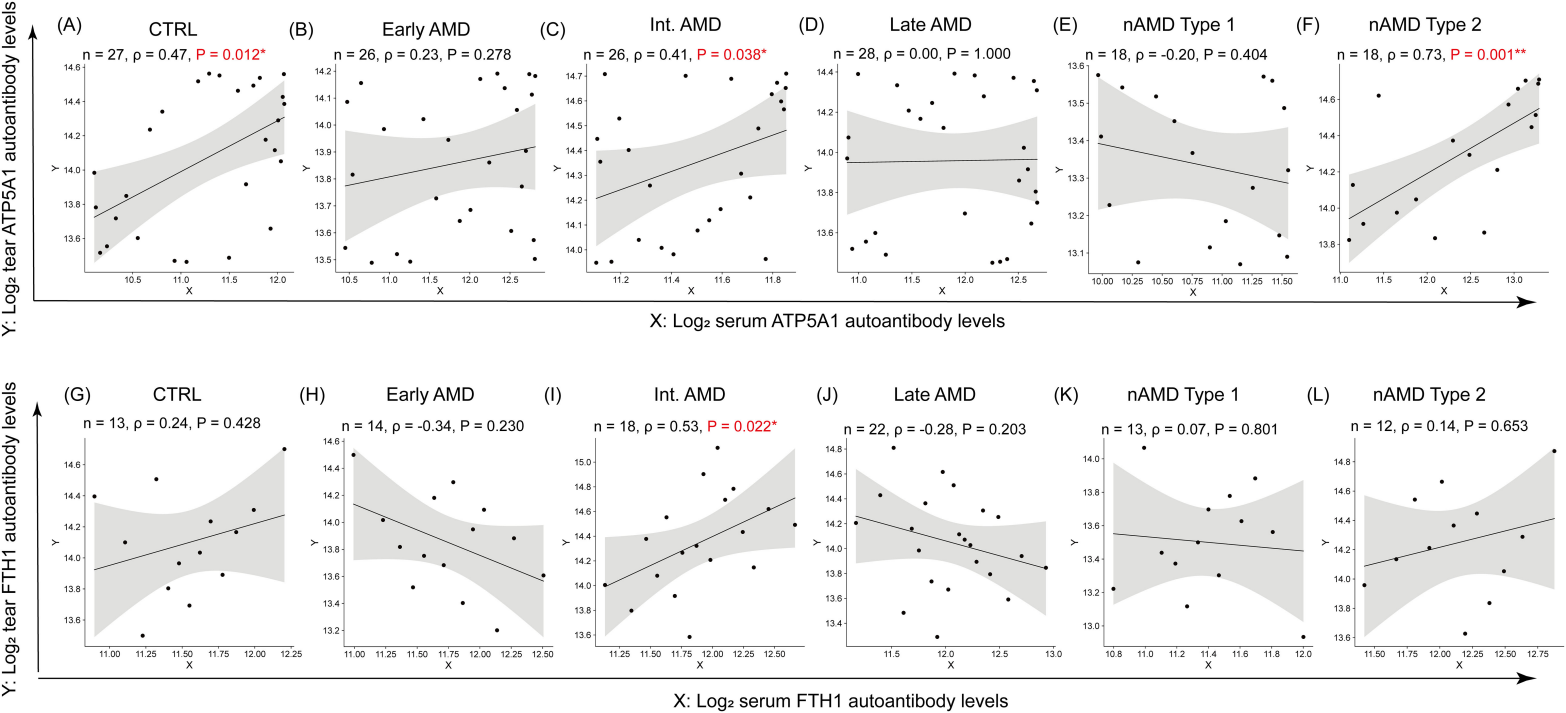
Correlation analysis between serum and tear autoantibody levels against selected retinal antigen targets in CTRL and AMD subgroups. Spearman correlation analysis was performed to assess the association between serum and tear autoantibody reactivity against ATP5A1 and FTH1. **(A–F)** Correlations between log_2_ serum and tear ATP5A1 autoantibody levels in CTRL, early AMD, Int. AMD, late AMD, nAMD Type 1, and nAMD Type 2 groups. **(G–L)** Correlations between log_2_ serum and tear FTH1 autoantibody levels in the corresponding groups. Each dot represents an individual sample. The black line indicates the fitted trend, and the shaded area represents the 95% confidence interval. Spearman’s rho (ρ), sample size (n), and p values are shown in each panel. Significant p values are highlighted in red.

As a subsequent analysis, Spearman rank correlation analysis was performed for four candidate anti-retinal autoantibody targets, including ATP5A1, FTH1, PFKM, and HIST2H2AA3, to assess the relationship between their levels in serum and tear samples. As shown in **Figure 7**, anti-ATP5A1 autoantibody levels showed significant positive correlations between serum and tears in healthy controls, intermediate AMD, and nAMD type 2, with Spearman correlation coefficients of 0.47, 0.41, and 0.73, respectively (p = 0.012, p = 0.038, and p = 0.001, respectively). Anti-FTH1 autoantibody levels also showed a significant positive correlation between serum and tears in intermediate AMD (Spearman ρ = 0.53, p = 0.022). In contrast, anti-PFKM and anti-HIST2H2AA3 autoantibody levels did not show significant serum–tear correlations in healthy controls or any AMD subgroup (all p > 0.05; data not shown).

## 4 Discussion

Early and effective screening may help reduce AMD-related visual impairment (17). Previous studies have reported that anti-retinal autoantibodies can be detected years before the onset of clinical symptoms in AMD patients (18). Therefore, investigating serum and tear immune reactivity across AMD stages may provide insight into disease-associated immune alterations and help clarify potential mechanisms involved in AMD progression. In this study, we systematically characterized serum autoantibody-captured retinal antigen targets across different AMD subtypes and further evaluated selected autoantibody responses in serum and tear samples.

Increasing evidence suggests that AMD may share neuroinflammatory and immune-related mechanisms with other neurodegenerative disorders, such as Alzheimer’s disease (19). Although the retina is considered an extension of the central nervous system, retinal and brain neurodegeneration are often investigated separately (19). In the present study, pathway enrichment and STRING network analyses of serum autoantibody-captured retinal antigen targets revealed prominent enrichment in modules related to neurodegeneration and mitochondrial function. These findings should not be interpreted as evidence of a direct clinical association between AMD and neurological diseases such as amyotrophic lateral sclerosis, Parkinson’s disease, or Huntington’s disease. Rather, they suggest that the identified retinal antigen targets may be involved in shared molecular processes associated with neurodegeneration, including neuronal stress, mitochondrial impairment, oxidative damage, and disrupted protein homeostasis. These shared mechanisms may represent potential overlapping pathogenic features or risk-related biological processes between AMD and other neurodegenerative diseases.

A notable subtype-specific finding was the prominent mitochondrial signature observed in nAMD type 1. Both pathway enrichment analysis and STRING network analysis highlighted proteins involved in mitochondrial respiration, electron transport chain activity, oxidative phosphorylation, ATP synthesis, and mitochondrial stress responses. This finding suggests that circulating autoantibodies in patients with nAMD type 1 may preferentially recognize retinal antigens associated with mitochondrial energy metabolism. In contrast, nAMD type 2 showed a broader interaction pattern involving the HIF-1 signaling pathway, mitochondrial function, glycolysis, and cytoskeleton-associated proteins. At present, intravitreal anti-VEGF therapy remains the main treatment for nAMD; however, subretinal fibrosis is a major contributor to anti-VEGF resistance and irreversible visual loss, affecting up to 67% of nAMD cases (20). Previous studies have implicated metabolic reprogramming (21), hypoxia (22), and mitochondrial dysfunction (23)as important initiating factors in the development of subretinal fibrotic membranes in wet AMD. Therefore, the retinal antigen targets identified in this study, particularly those associated with HIF-1 signaling, mitochondrial function, glycolysis, and structural remodeling, may provide useful clues for further understanding the immunological features associated with nAMD pathogenesis and subretinal fibrosis.

Interestingly, several significantly altered retinal antigen targets were shared across different AMD stages, whereas others appeared to be more stage restricted. For example, some antigenic changes were observed in early and intermediate AMD, while others persisted from early disease into nAMD type 1, emerged from late non-neovascular AMD toward nAMD type 2, or were restricted to neovascular AMD. This pattern suggests that the anti-retinal autoantibody profile in AMD is not static but may undergo dynamic remodeling during disease progression and neovascular transformation. To further explore whether selected MS-derived retinal antigen targets correspond to measurable autoantibody responses in ocular fluid or serum, we performed targeted microarray analysis in tear and serum samples. Among the dynamically altered candidates, autoantibody responses against ATP5A1, PFKM, HIST2H2AA3, and FTH1 showed changes that were further supported by microarray analysis. These findings provide partial validation of the proteomics-based screening results.

Oxidative stress and mitochondrial dysfunction are central pathological features of AMD (24). ATP5A1, a key component of the mitochondrial oxidative phosphorylation complex V (24), was more frequently detected in neovascular AMD than in non-neovascular AMD. This finding may suggest that vascular endothelial injury and increased cell death in neovascular AMD could promote the release of mitochondrial proteins, such as ATP5A1, into the extracellular environment, thereby triggering immune recognition (25). Therefore, elevated anti-ATP5A1 autoantibody levels may represent an indirect indicator of mitochondrial injury severity in AMD, although this interpretation requires further validation.

PFKM, the rate-limiting enzyme of glycolysis (26), plays an essential role in high-energy–demand or hypoxic conditions. Its corresponding autoantibody was significantly elevated in serum and tears of neovascular AMD type 2 patients, suggesting that metabolic reprogramming—particularly increased glycolytic flux—occurs in RPE and choroidal endothelial cells under hypoxia-related stress. This finding aligns with the current understanding of metabolic dysregulation in neovascular AMD (27).

HIST2H2AA3, a member of the core histone H2A family, is normally confined to the nucleus (21). Increased extracellular release due to enhanced apoptosis or necrosis can act as a damage-associated molecular pattern, activating TLR2/TLR4 and complement pathways. The elevated anti-HIST2H2AA3 autoantibodies observed in this study likely reflect increased cell death and inflammation in AMD (21).

FTH1 is a major iron-storage protein that helps regulate intracellular labile iron homeostasis (28). Reduced FTH1 expression may increase the availability of free iron, thereby promoting oxidative stress through the Fenton reaction and contributing to ferroptosis (29). Downregulation of FTH1 has also been regarded as a feature associated with ferroptotic cell death (30). Ferroptosis is increasingly recognized as an important mechanism contributing to RPE degeneration in AMD (31). In the present study, anti-FTH1 autoantibody levels were significantly decreased in nAMD type 1, suggesting that altered immune recognition of FTH1-related retinal antigens and disturbed iron homeostasis may be involved in this AMD subtype.

Importantly, we report for the first time an increase in tear anti-SAG (S-antigen) autoantibodies across different AMD subtypes, with the greatest increase in neovascular AMD type 2. SAG is a classical retinal autoantigen (32) that plays a pathogenic role in autoimmune related retinopathy (33) and experimental autoimmune related uveitis (32). Its release during photoreceptor damage may account for the development of circulating and tear autoantibodies (34). The presence of anti-SAG autoantibodies in tears indicates a potential association between local anti-retinal autoantibody responses and photoreceptor-related antigen recognition in AMD, although its clinical relevance requires further validation.

This study has several strengths. First, we performed a comprehensive proteomics-based screening of serum autoantibody-captured retinal antigen targets across five AMD stages, providing insight into the immune heterogeneity and dynamic changes associated with different AMD subtypes. Compared with conventional serum autoantibody profiling, our approach focused on autoantibodies that specifically bound to retinal antigens from porcine retinal homogenates, which may help reduce interference from systemic, non-retina-related autoantibodies (35).

However, several limitations should also be acknowledged. First, sequence differences between human and porcine retinal proteins may have resulted in incomplete antigen capture and potential false-negative findings (36). Second, only a selected subset of retinal antigen targets identified by mass spectrometry was further evaluated by targeted antigen microarray analysis. Therefore, the microarray results should not be interpreted as independent external validation or comprehensive validation of all MS-derived candidates, and more extensive validation will be required in future studies. Third, because this was a cross-sectional study, it cannot determine whether the observed autoantibody responses contribute to AMD pathogenesis or merely represent secondary immune responses following retinal damage and antigen exposure. Fourth, although this study included different AMD subtypes, the sample size of some subgroups was relatively small, with certain subgroups including only approximately 10 participants. This may limit statistical power, increase the risk of unstable effect estimates or false-positive finding. Fifth, tear composition may be influenced by multiple ocular surface-related factors. Although participants with other ocular diseases, particularly dry eye disease, meibomian gland dysfunction, ocular surface infection, and other clinically evident ocular surface disorders, were excluded as far as possible, residual confounding from ocular surface status, reflex tearing, tear volume variation, or pre-analytical factors cannot be completely ruled out. Finally, although we observed differences in serum and tear anti-retinal autoantibody responses across AMD stages, no formal biomarker performance analysis or predictive model was established, including receiver operating characteristic curve analysis, area under the curve estimation, sensitivity and specificity assessment, internal validation, or external validation. Therefore, the observed differences in serum and tear anti-retinal autoantibodies should be interpreted as exploratory and hypothesis-generating findings rather than clinically applicable diagnostic or prognostic biomarkers. Further validation using human retinal antigens, larger independent cohorts, and longitudinal samples will be required to confirm the biological and clinical relevance of these findings.

## 5 Conclusion

In conclusion, this exploratory immunoproteomic study characterized serum autoantibody-captured retinal antigen targets across AMD subtypes. The observed disease-stage-associated autoantibody patterns, supported by pathway enrichment, STRING network analysis, and targeted serum and tear microarray validation of selected retinal antigen targets, suggest that anti-retinal autoantibody responses may reflect distinct immunological features during AMD progression. Among the retinal antigen targets identified by mass spectrometry, autoantibody responses against FTH1, ATP5A1, PFKM, and HIST2H2AA3 showed concordant changes in targeted serum and/or tear microarray analysis, particularly in neovascular AMD subtypes. Overall, these findings may provide insight into stage-associated immune alterations in AMD and highlight selected retinal antigen targets that warrant further validation in larger independent cohorts.

## Data Availability

The raw data supporting the conclusions of this article will be made available by the authors, without undue reservation.
