# Supplementary material for: Stage-associated immunoproteomic profiling of serum autoantibody-captured retinal antigens in age-related macular degeneration
Source: Front Immunol. 2026 Jul 8;17:1824524. doi: 10.3389/fimmu.2026.1824524 (PMC13388467; doi:10.3389/fimmu.2026.1824524)
Supplement: Supplementary file 1 [file Table1.docx]

Supplementary Methods

**AMD Clinical Classification**

**Early AMD：**Medium drusen >63 μm and ≤125 μm and No AMD pigmentary abnormalities* .

**Intermediate AMD：**Large drusen > 125 μm and/or Any AMD pigmentary abnormalities* .

**Late AMD：**Any geographic atrophy.

**Type 1 neovascular AMD** was defined as macular neovascularization located beneath the retinal pigment epithelium, within the sub-RPE space between the retinal pigment epithelium and Bruch’s membrane.

**Type 2 neovascular AMD** was defined as macular neovascularization extending above the retinal pigment epithelium into the subretinal space.

*AMD pigmentary abnormalities = any definite hyper- or hypopigmentary abnormalities associated with medium or large drusen but not associated with known disease entities.

AMD grading was performed according to a previously published consensus classification system using available multimodal retinal imaging data. Grading was primarily based on color fundus photography and spectral-domain optical coherence tomography (SD-OCT), with fluorescein angiography additionally reviewed when necessary, particularly for confirming neovascular AMD and determining neovascularization subtype. Images were anonymized, coded, and evaluated independently by two trained graders who were masked to proteomics, antigen microarray, and other laboratory results. In cases of disagreement, the images were re-evaluated jointly and resolved by consensus. Persisting discrepancies were adjudicated by a senior retinal specialist.

**List of inclusion and exclusion criteria.**

**Patients:**

Participants were considered eligible for inclusion if they met all of the following criteria: newly diagnosed AMD; male or female sex; age ≥50 years; absence of other concomitant ocular diseases, including ocular surface diseases (dry eye disease, meibomian gland dysfunction, ocular surface infection); absence of hematological disorders; absence of systemic diseases, including acute infectious diseases, autoimmune diseases, metabolic syndrome, malignancies, or other relevant systemic conditions; no history of intraocular surgery within the previous 2 months; no use of systemic medications or ocular medications within the previous 3 months; and the ability to understand the nature of the clinical study and its personal implications.

Written informed consent, signed and dated by the participant, was obtained before the initiation of any study-specific procedures.

**Healthy volunteers**

Healthy volunteers were considered eligible for inclusion if they met all of the following criteria: male or female sex; age ≥50 years; absence of ocular diseases, including ocular surface diseases (dry eye disease, meibomian gland dysfunction, ocular surface infection); absence of hematological disorders; absence of systemic diseases, including acute infectious diseases, autoimmune diseases, metabolic syndrome, malignancies, or other relevant systemic conditions; ability to understand the nature of the clinical study and its personal implications; no history of intraocular surgery within the previous 2 months; and no use of systemic medications or ocular medications within the previous 3 months.

Written informed consent, signed and dated by the participant, was obtained before the initiation of any study-specific procedures.

**Statistical analysis and bioinformatics analysis**

Differentially captured retinal antigens were identified by comparing each AMD subgroup with the CTRL group, including early AMD vs. CTRL, Int. AMD vs. CTRL, Late AMD vs. CTRL, nAMD type 1 vs. CTRL, and nAMD type 2 vs. CTRL. Group-wise comparisons were performed using two-tailed Student’s t-tests. P values derived from these comparisons were adjusted for multiple testing using the Benjamini–Hochberg false discovery rate method. Proteins with p < 0.05 and FDR < 0.05 were considered significantly differentially captured retinal antigen targets in the discovery proteomics analysis. Log₂ fold changes were calculated based on the difference in mean log₂-transformed LFQ intensities between each AMD subgroup and CTRL.

Heatmaps, Venn diagrams, and GO/pathway enrichment visualizations were generated using the online bioinformatics platform Bioinformatics.com.cn (http://www.bioinformatics.com.cn/). Hierarchical clustering in heatmaps was performed using Euclidean distance to visualize group-specific abundance patterns of the differentially captured retinal antigens. Functional enrichment analysis was performed using the differentially captured retinal antigen targets identified in each AMD subgroup compared with CTRL. Gene Ontology biological process and pathway enrichment results were visualized as bubble plots, in which the enrichment score was calculated as −log₁₀ (p value), bubble size represented the number of proteins involved in each term or pathway, and color represented the corresponding p value. Protein–protein interaction network analysis was performed using the STRING database (https://string-db.org/). Differentially captured retinal antigen targets from each AMD subgroup were submitted to STRING to explore known and predicted functional interactions. STRING networks were used to identify potential functional clusters and interaction modules among the candidate retinal antigen targets.

For the targeted antigen microarray validation phase, serum and tear autoantibody reactivities against selected MS-derived retinal antigen targets were analyzed. Raw fluorescence signals were extracted after exclusion of technically flawed spots. Background-corrected mean intensities were normalized using a constant scaling factor for each subarray, and technical triplicates were averaged before statistical analysis. Statistical analyses of antigen microarray data were performed using Statistica software (version 13). Overall differences among CTRL and AMD subgroups were first assessed using the Kruskal-Wallis test. Pairwise comparisons between CTRL and individual AMD subgroups were then performed using Mann–Whitney U tests. P values < 0.05 were considered statistically significant. Because the antigen microarray analysis was performed on a selected subset of candidate retinal antigens identified from the discovery proteomics phase, these validation analyses were interpreted as exploratory.

For serum–tear association analysis, Spearman rank correlation was used to assess correlations between serum and tear autoantibody levels against selected candidate retinal antigen targets. Spearman’s correlation coefficient, sample size, and p value were reported for each comparison. All statistical tests were two-sided unless otherwise stated.
