# Supplementary material for: Stage-associated immunoproteomic profiling of serum autoantibody-captured retinal antigens in age-related macular degeneration
Source: Front Immunol. 2026 Jul 8;17:1824524. doi: 10.3389/fimmu.2026.1824524 (PMC13388467; doi:10.3389/fimmu.2026.1824524)
Supplement: Supplementary file 2 [file Table2.docx]

Supplementary Material

**Table S1: list of antigens used for microarray analysis**

| **Recombinant protein name** | **Gene name** | **Suppliers** | **Cat. No.** |
| --- | --- | --- | --- |
| Recombinant Human PFKM Protein | PFKM | Abcam | AB95304 |
| Recomb Hunman ATP5A1 Protein | ATP5A1 | enQuirebio | QP5693-EC-100UG |
| Recombinant Human Histone H2A Protein | HIST2H2AA3 | Abcam | AB200295 |
| PSMD6 Recombinant Protein Antigen | PSMD6 | bio-techne | NBP1-81545PEP |
| HSPA12A Recombinant Protein Antigen | HSPA12A | bio-techne | NBP2-57850PEP |
| PRMT5 Recombinant anti protein antigen | PRMT5 | bio-techne | NBP1-81701PEP |
| Recombinant Human p23/PTGES3 Protein | PTGES3 | Novus Biologicals | NBP3-18323 |
| SEC22B protein,Recombinant Human aa14-194 | SEC22B | USBiological | l24101501 |
| Recombinant Human Ferritin Heavy Chain protein | FTH1 | Abcam | ab78877 |
| S-Arrestin Recombinant Protein | SAG | My Biosource | MBS955104 |
| Succinate Dehydrogenase Complex Subunit B, Partial Recombinant Protein | SDHB | My Biosource | MBS2031006 |
| Recombinant Human Vimentin protein | VIM | Abcam | ab84704 |
